# Amblyopinae Mitogenomes Provide Novel Insights into the Paraphyletic Origin of Their Adaptation to Mudflat Habitats

**DOI:** 10.3390/ijms24054362

**Published:** 2023-02-22

**Authors:** Zhenming Lü, Yantao Liu, Shijie Zhao, Jiaqi Fang, Kehua Zhu, Jing Liu, Li Gong, Liqin Liu, Bingjian Liu

**Affiliations:** 1 National Engineering Laboratory of Marine Germplasm Resources Exploration and Utilization, College of Marine Sciences and Technology, Zhejiang Ocean University, Zhoushan 316000, China; 2 National Engineering Research Center for Facilitated Marine Aquaculture, Zhejiang Ocean University, Zhoushan 316000, China

**Keywords:** Amblyopinae mitogenome, paraphyletic origin, mudflat habitat adaptation

## Abstract

The water-to-land transition is one of the most important events in evolutionary history of vertebrates. However, the genetic basis underlying many of the adaptations during this transition remains unclear. Mud-dwelling gobies in the subfamily Amblyopinae are one of the teleosts lineages that show terrestriality and provide a useful system for clarifying the genetic changes underlying adaptations to terrestrial life. Here, we sequenced the mitogenome of six species in the subfamily Amblyopinae. Our results revealed a paraphyletic origin of Amblyopinae with respect to Oxudercinae, which are the most terrestrial fishes and lead an amphibious life in mudflats. This partly explains the terrestriality of Amblyopinae. We also detected unique tandemly repeated sequences in the mitochondrial control region in Amblyopinae, as well as in Oxudercinae, which mitigate oxidative DNA damage stemming from terrestrial environmental stress. Several genes, such as *ND2*, *ND4*, *ND6* and *COIII*, have experienced positive selection, suggesting their important roles in enhancing the efficiency of ATP production to cope with the increased energy requirements for life in terrestrial environments. These results strongly suggest that the adaptive evolution of mitochondrial genes has played a key role in terrestrial adaptions in Amblyopinae, as well as in Oxudercinae, and provide new insights into the molecular mechanisms underlying the water-to-land transition in vertebrates.

## 1. Introduction

The water-to-land transition is one of the most important events in evolutionary history, as it led to an explosive radiation of tetrapods, which is the most successful group of vertebrates on land [1]. Previous studies have provided insights into how tetrapods successfully moved onto land after the ancestor of bony fishes first colonized land during the Paleozoic era [2,3]. Interestingly, several groups of bony fishes that emerged much later also independently evolved terrestrial adaptations that enabled them to spend a considerable part of their life on land [4,5]. These adaptations often include morphological, behavioral, and physiological changes that allow organisms to cope with the challenges of terrestrial environments, including hypoxia, temperature variation, and low, often fluctuating humidity [1,4,5]. However, little is known about the genetic basis of these adaptations. 

Eel gobies (family Gobionellidae; subfamily Amblyopinae) are the largest group of marine fishes showing unique adaptations to life in terrestrial habitats [6,7,8,9]. Typically, they live in burrows in tidal mudflats and the muddy bottoms of estuaries, but they can also be found in trawls of muddy substrates from the sea up to approximately 100 m in depth [9]. Some species (e.g., the genus *Odontamblyopus* and *Taenioides*) are so well adapted to terrestrial environments that they possess unique abilities to breathe air via their richly vascularized inner epithelia in the buccal-opercular cavity to cope with the hypoxia conditions in their tidal mudflat habitat [6,8]. Therefore, eel gobies may represent one of the lineages of bony fishes that shows the primitive terrestrial adaptation and are thus a useful group for clarifying the genetic changes underlying terrestrial adaptations during the early water-to-land transition. Despite substantial work on the morphological, behavioral, and physiological characteristics of eel gobies [6,10,11], few studies have examined the molecular basis underlying adaptations to terrestrial mudflat habitats in these species, or in other non-Amblyopinae teleost fishes. It is, therefore, interesting to investigate the genetic mechanisms underlying their adaptations to mudflat habitats. 

Mitochondrial energy metabolism plays an important role in mediating adaptation to the terrestrial environment by aquatic animals through aerobic respiration [12]. The adenosine triphosphate (ATP) produced by mitochondria can supply energy for life activities and help maintain physiological homeostasis in unstable environments [13]. The mitochondrial genome contains 13 protein-coding genes (PCGs) that encode proteins involved in aerobic respiration [12]. To adapt to the unstable terrestrial environments, aerobic respiration in aquatic animals needs to undergo natural selection to improve their ATP production efficiency. Therefore, the evolution of mitochondrial genes might be affected by the terrestrial environment. Indeed, several mitochondrial DNA (mtDNA) analyses have detected signatures of adaptive evolution in the mitochondrial genes of terrestrial panpulmonate gastropods [12], crayfishes [14], and chitons [15]. However, evolutionary changes underlying the adaptations of Amblyopinae to terrestrial mudflat habitats have never been investigated.

Here, we sequenced the complete mitogenome sequences of six Amblyopinae species and compared them with those of their closest terrestrial and non-terrestrial relatives to clarify the molecular basis underlying adaptation to terrestrial habitats in bony fishes during the early water-to-land transition. Given that signatures of adaptive evolution have been observed in the mitochondrial genes of other terrestrial marine animals [12,15], we hypothesized that positive selection will also be observed in the oxidative phosphorylation (OXPHOS) related mitochondrial genes in mud-dwelling Amblyopinae.

## 2. Results

### 2.1. Characteristics of Amblyopinae Mitogenomes

**Figure 1:**
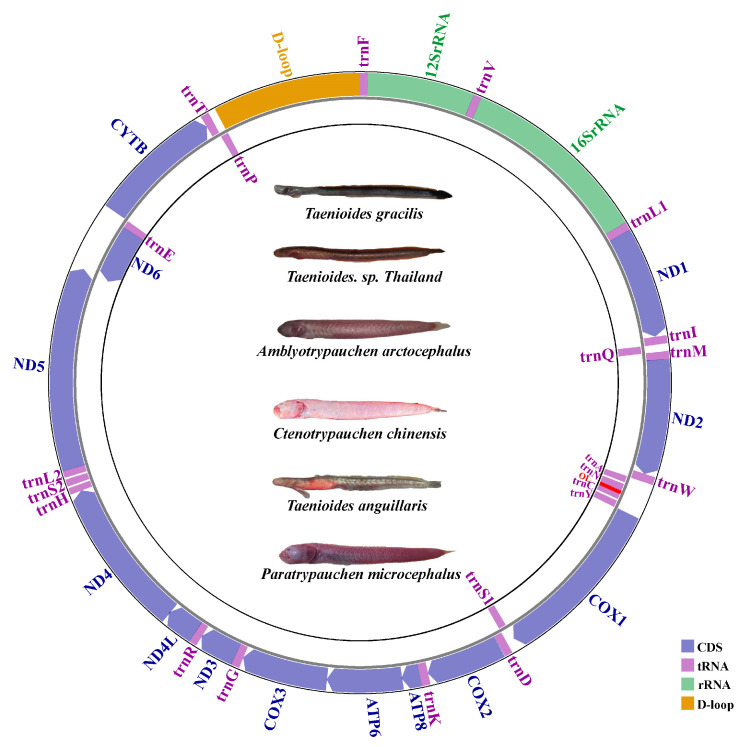
The gene organization of the mitochondrial genome of six Amblyopinae species. All protein-coding genes are encoded on the H-strand, with the exception of ND6, which is encoded on the L-strand. The two ribosomal RNA genes are encoded on the H-strand. Transfer RNA genes are designated by single-letter amino acid codes. Genes encoded on the H-strand and L-strand are shown outside and inside the circular gene map, respectively.

**Figure 2:**
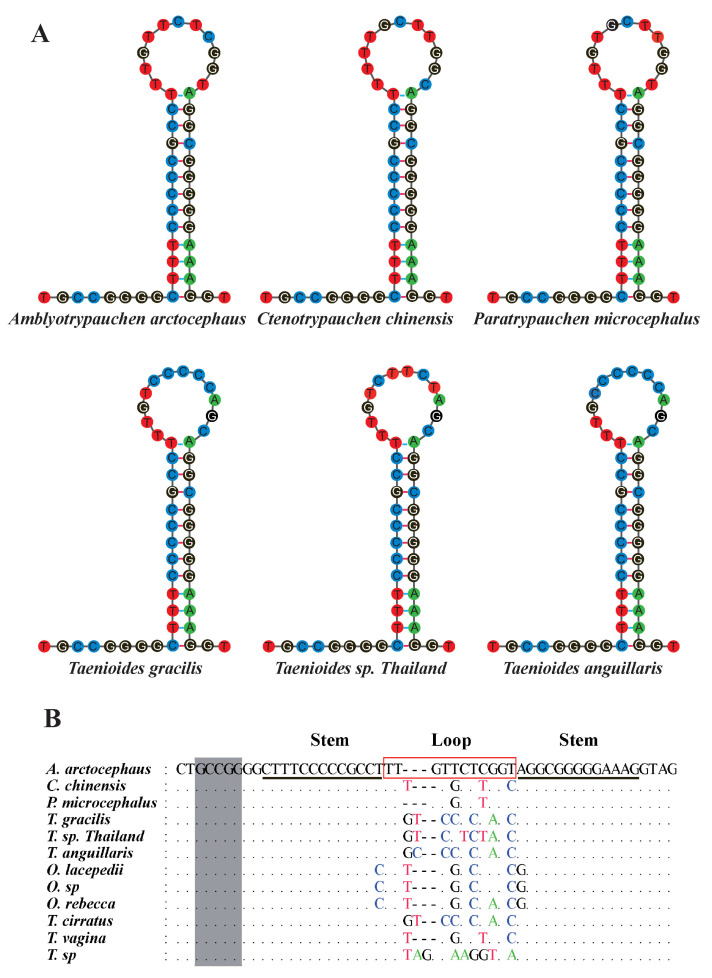
(**A**) Sequence alignment of OL of six Amblyopinae species. The highly conserved sequence motif (-GCCGG), which was found at the base of the stem within tRNA-Cys, is marked by the grey shading. The region, which is underlined with a black line and the red box, indicates the stem and loop of the putative stem-loop secondary structures, respectively. Identical nucleotides are denoted by dots, and replacements are indicated by the corresponding nucleotides; dashes represent indels. (**B**) The putative stem-loop secondary structures of six Amblyopinae species. The sequences are presented as H-strand sequence.

We sequenced six new Amblyopinae mitogenomes. The general characteristics of the mitogenomes are summarized in Table 1 and Supplementary Tables S3–S8. The length of the mitogenomes generally ranges from 16,552 bp to 17,133 bp. All the mitogenomes encode 13 PCGs, 2 rRNAs, 22 tRNAs, and a putative control region, as has been reported for most other animal mitogenomes. Most genes are encoded on the heavy strand, only the gene encoding the NADH dehydrogenase subunit 6 (ND6) and eight tRNA (*Ala, Asn, Cys, Gln, Glu, Pro, Ser*, and *Tyr*) genes are encoded on the light strand. The arrangement of genes is similar to that observed in the mitogenomes of other goby species. We characterized the main features of the mitogenomes in Amblyopinae using these six new mitogenomes, as well as six other mitogenomes deposited in Genbank. A noteworthy feature of the Amblyopinae mitogenomes is the 50–53 bp putative origin of L-strand replication (OL) located between tRNA-Asn and tRNA-Cys in a cluster of five tRNA genes (*Ala*, *Asp*, *Cys*, *Trp*, and *Tyr*), which is known as the WANCY region [16] (Figure 1). It forms a stem-loop secondary structure (Figure 2A), which is a general characteristic of the origin of light strand replication. The highly conserved sequence motif 5′-GCCGG-3′, which is involved in the transition from RNA to DNA synthesis, was observed at the base of the stem within tRNA-Cys (Figure 2B). Another feature of Amblyopinae mitogenomes is the non-coding control region located between tRNA-Pro and tRNA-Phe, which contains tandemly repeated sequences (TRS). Both perfect and imperfect repeats were observed in the TRS (Table 2), as in other animal mitogenomes. Both the number (2–6 times) and the length (120–163 bp) of the tandem repeats varied greatly among species, which might imply a rapid evolution of the structure in the control region. However, the starting sequences (-AAACAGGA) of the tandem repeats in the control region were generally conserved among species, with the exception of species in the genus *Odontamblyopus*, in which the sequences start more than 140 bp behind in the mitogenome (started with -AAAGATTT) (Table 2), possibly indicating their different evolutionary origins. 

### 2.2. Phylogenetic Analyses

**Figure 3:**
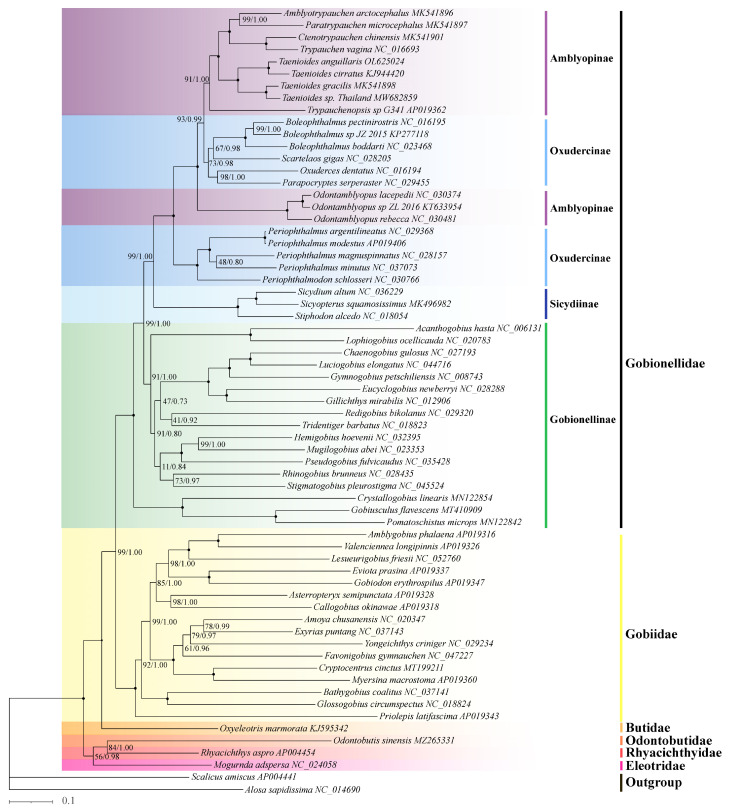
The phylogenetic relationship between Amblyopinae and their closely related species in Gobioidei. The phylogeny was inferred from the amino acid sequences of 13 PCGs of the 63 Gobioidei mitogenomes examined using both Bayesian inference (BI) and maximum likelihood (ML) methods. *Scalicus amiscus* and *Alosa sapidissima* were used as the outgroups. The numbers on the branches are posterior probability (left) for Bayesian inference and bootstrap support (right) for ML analyses.

**Figure 4:**
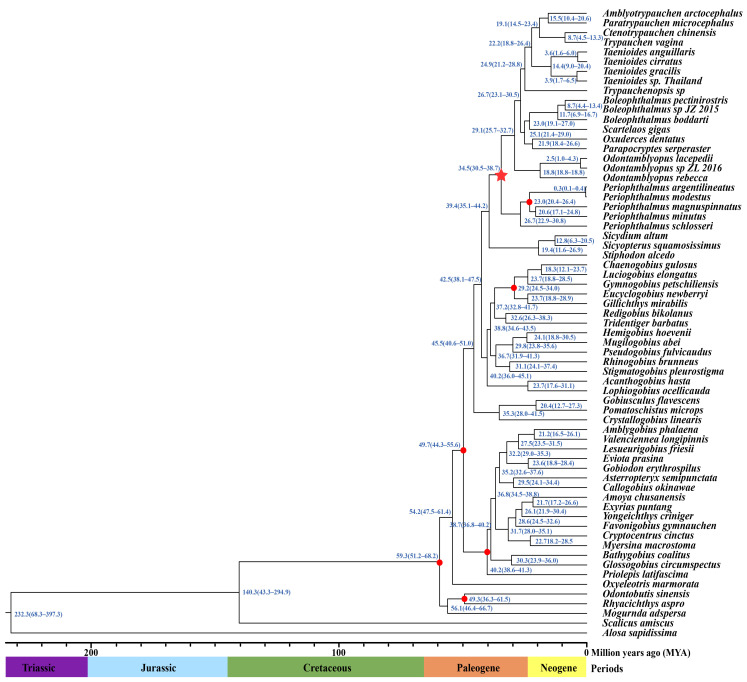
Time tree constructed for the Amblyopinae species. The tree topology derived from these fishes is generally consistent with the Bayesian inference shown in Figure 3. Branch lengths are proportional to divergence times. The numbers at the right of the nodes are the estimates of the mean divergence times (in My). The red circle indicates the fossil record used for calibration in the node. The red star indicates the time estimated for the emergence of paraphyletic clade of terrestrial gobies (Amblyopinae + Oxudercinae).

We constructed the phylogeny of Amblyopinae using concatenated sequences of 13 coding sequences (CDSs) using our six newly sequenced mitogenomes and 57 published mitogenomes sequences from Gobioidei. The mitogenomic phylogenetic analyses yielded trees with consistent topologies and with high levels of support based on both ML and BI inference methods (Figure 3). Both the ML and BI trees indicated that species in the genera *Amblyotrypauchen*, *Paratrypauchen*, *Ctenotrypauchen*, *Trypauchen*, and *Taenioides* were more closely related to non-Amblyopinae species in the subfamily Oxudercinae than to species in the genus *Odontamblyopus* comprising their sister clade. The observation that, in both trees, species in genera *Amblyotrypauchen*, *Paratrypauchen*, *Ctenotrypauchen*, *Trypauchen*, and *Taenioides* are clustered with non-Amblyopinae species rather than Amblyopinae species in the genus *Odontamblyopus* provides strong support for the paraphyletic origins of Amblyopinae. This suggests that these two subfamilies should be merged and reveals an expansion of phenotypic variation within the “terrestrial goby” clade, as has been suggested by Steppan [22]. Using fossil calibration, we estimated that this paraphyletic clade emerged approximately 34.5 million years ago (My) in the late Paleogene (Figure 4). However, within the clade, species in the genus *Periophthalmus* appear to have evolved slightly earlier (34.5 My vs. 29.1 My) than the rest of the species, which indicates that they comprise a basal lineage in terrestrial gobies. 

### 2.3. Positive Selection Analyses

**Figure 5:**
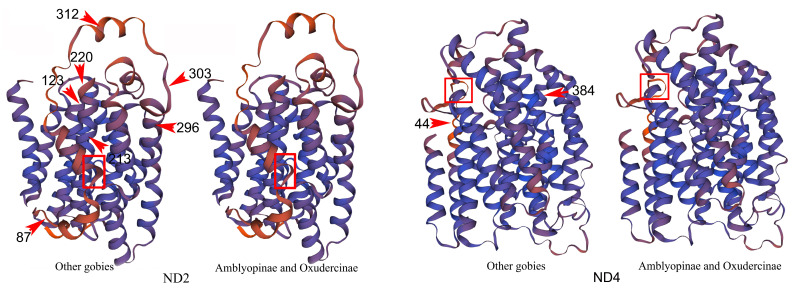
The predicted three-dimensional protein structure of ND2 and ND4 in Amblyopinae and Oxudercinae, as well as in their relatives. Residues with amino acid under selection as determined by PMAL are shown in the figures. Arrows point to these mutations and the numbers show the position of changes. The changes in the three-dimensional structure of ND2 and ND4 brought by these positively selected sites are marked by the red squares.

The selection pressure on mtDNA genomes was evaluated using CODEML in PAML v4.8a software, and the results of the analysis are shown in Table 3. The average ω ratio for each of the 13 PCGs calculated from M0 in the branch-specific model was significantly less than 1, suggesting that all the mitochondrial genes in the sampled Amblyopinae species and their terrestrial relatives in Oxudercinae have evolved under strong functional constraints, which is consistent with the known functional significance of mitochondria as respiration chains necessary for OXPHOS and electron transport. However, based on the two-ratio (M2) model, where the paraphyletic clade of terrestrial gobies (Amblyopinae + Oxudercinae) was set as the foreground, and the other non-terrestrial gobies within the same family of Gobionellidae were set as the background, we found that seven PCGs (*COI*, *COIII*, *ND1*, *ND2*, *ND4*, *ND5*, and *ND6*) of the clade had significantly (*p* < 0.05) higher ω values than other Gobioidei species, which indicates that these genes have been positively selected in terrestrial gobies (Table 3). The branch-site model was further used to detect positive selection in individual codons. Our analyses suggested that there was significant evidence of positive selection along the branch leading to the paraphyletic clade of terrestrial gobies. Eleven residues in the four PCGs, *COIII* (G162S), *ND2* (Q87T, D123T, S213A, F220N, C296S, M303I, S312T), *ND4* (N44S, T384V), and *ND6* (Y78F) were inferred as positively selected sites in the terrestrial goby branch with posterior probabilities greater than 95% (Table 4). A high proportion (54.55%) of changes in amino acids resulting in changes in the property of proteins, including their polarity or hydrophilicity, were detected in branches leading to species that inhabit mudflats (Supplementary Table S9). The three-dimensional structural model of the proteins encoded by these four mtDNA genes showed that selection has affected the structure of ND2 and ND4 in complex I of the mitochondrial respiratory chain (Figure 5). The observed evolutionary changes in both amino acid properties and protein structure might imply functional alterations of mitochondria in terrestrial gobies that enhance aerobic respiration in mudflat habitat. 

## 3. Discussion

The mitogenomes in animals contain 13 PCGs, and their products are necessary for oxygen usage and energy metabolism. An increasing number of cases of adaptive evolution in mitochondrial genes have been reported in aquatic animals [12,14,15]. In the present study, we analyzed the whole mitogenomes of six species in the subfamily Amblyopinae, along with the 57 mitogenomes of Gobioidei species deposited in Genbank, to detect signs of adaptive evolution on OXPHOS genes. 

We did not detect signs of gene re-arrangements of mitogenomes in Amblyopinae. The mitogenomes of Amblyopinae were similar in size and gene arrangement to those of other Gobioidei species. No obvious expansion, contraction, or new gene arrangements were observed in Amblyopinae mitogenomes, yet such changes have often been observed in animals that inhabit harsh environments [23,24,25]. TRS were common in the control regions of the Amblyopinae mitogenomes. TRS were also frequently observed in Oxudercinae species (Table 2), as well as in other animals inhabiting abiotic stress environment [24,25,26]. The occurrence of TRS in the control region was inferred to provide advantages in mitochondrial DNA replication because they provide an “attractive” conformation that permits the more efficient binding of DNA polymerase [27]. The high efficiency of mtDNA replication is necessary for compensating for the oxidative damage to DNA associated with environmental stress [27]. Therefore, TRS are more common in species inhabiting harsh environments [24,25,26]. Our results support this expectation, given that Amblyopinae and Oxudercinae species might require high mtDNA replication efficiency to compensate for the potential oxidative damage to DNA induced by the unstable oxygen, temperature, and low, often fluctuating moisture in mudflat habitats. 

To detect the signs of adaptive evolution acting on OXPHOS genes, we first constructed the phylogenetic trees of Amblyopinae based on the ML and BI inference methods. The result yielded trees with consistent topologies, and all branches were highly supported. In contrast to the monophyly inferred from traditional taxonomy of subfamily Amblyopinae, both the ML and BI trees strongly supported the paraphyletic origins of Amblyopinae with respect to Oxudercinae, which is consistent with the results obtained from several recent studies using both nuclear and mitochondrial DNA [22,28,29]. Additional analyses of the non-coding control regions revealed that the genera *Amblyotrypauchen*, *Paratrypauchen*, *Ctenotrypauchen*, *Trypauchen*, and *Taenioides* in Amblyopinae generally shared the same starting sequences (-AAACAGGA) of TRS with some Oxudercinae species, rather than with the genus *Odontamblyopus*, which again supports a paraphyletic origin of Amblyopinae (Table 2). These findings indicate that these two subfamilies should be merged, as has been previously suggested by Steppan [22]. However, the paraphyly of Amblyopinae with respect to Oxudercinae reveals an interesting evolutionary scenario among members of Gobioidei because, despite their substantial differentiation in phenotype and physiology, the more aquatic “eel-like” mud-dwelling Amblyopinae and the more terrestrial mudskipper Oxudercinae may have evolved terrestrial behavior from their common ancestors. The fact that species in the genus *Odontamblyopus* are more closely related to non-Amblyopinae species in Oxudercinae, and species in the genus *Periophthalmus* form a sister clade suggests that two independent water-to-terrestrial transitions have occurred in this lineage, or alternatively, the more aquatic Amblyopinae may have evolved from Oxudercinae through a “secondary loss” of their terrestrial traits. Although the slightly earlier emergence of *Periophthalmus* (34.5 My vs. 29.1 My) estimated for the lineage appears to support the latter, more detailed analyses are needed to clarify the evolutionary scenarios in these paraphyletic clades. 

Our results from both the branch and branch-site models revealed strong evidence of positive selection on the ancestral branch leading to the paraphyletic clade of terrestrial gobies (Table 3 and Table 4). The results from the branch model showed that seven PCGs (*COI*, *COIII*, *ND1*, *ND2*, *ND4*, *ND5*, and *ND6*) in these paraphyletic clades had significantly higher ω values (*p* < 0.05) than other Gobioidei species, suggesting a signal for positive selection (Table 3). Furthermore, our results from the branch-site model revealed 11 positively selected residues in four PCGs (*COIII*, *ND2*, *ND4*, and *ND6*) with posterior probabilities greater than 95%, further suggesting that the positive selection has acted on these genes in this paraphyletic clade (Table 4). These results are not surprising given that both cytochrome oxidases and NADH dehydrogenases play important roles in aerobic metabolism and an ever-increasing number of studies has shown that these mitochondrial genes could be targets of positive selection [12,15]. Intriguingly, our analyses have revealed a high concentration of evolutionary changes caused by positive selection pressure acting on the mitochondrial lineages of terrestrial gobies which might reflect mitochondrial adaptation to physiological stress in mudflat environments. Therefore, positive selection might be one of the major forces driving the evolution of mitochondrial OXPHOS genes to cope with environmental change in teleosts, as has been observed in other animals [12,15]. 

NADH dehydrogenase is the largest enzyme complex (OXPHOS complex I) in the mitochondrial electron transport chain [30,31], and it serves as a proton pump that mediates the transport of H+ ions from the matrix to the inner membrane space, which drives ATP production. Mutations in this complex likely affect the efficiency of proton pumping and thus metabolic efficiency. Our results indicated that the majority of the sites under positive selection were in this complex, especially the three genes *ND2*, *ND4*, and *ND6*, which indicates that ND genes have experienced strong selection. The gene that has experienced the strongest selection is ND2, which is a common target of positive selection in diverse taxa inhabiting harsh environments [15,32,33]. We identified seven positively selected sites in this gene, and six were present in or near the loop regions instead of in the transmembrane domain (Figure 5). High concentrations of positively selected sites in the loop regions of the mitogenome-encoded proteins have previously been observed in mammals [31] and birds [34,35] and this is interpreted as a result of relaxation of functional constraints on these regions compared with transmembrane domains [31,34]. However, Fiedorczuk [36] and Zhu [37] revealed that residues in loop regions may be critical for interactions with the supernumerary subunits of OXPHOS complex I, therefore, changes in these regions might have major effects on proton translocation pathways or protein–protein interactions mediated by these subunits [35]. We also identified positively selected sites in *ND4*, which is another gene that encodes elements of proton pumps in the hydrophobic region of complex I. This is also consistent with the results of a previous study examining chiton inhabiting the intertidal zone [15], which showed signals of positive selection in this proton pumping gene. Some studies have revealed mutations in other *ND* genes that are linked to adaptations in harsh environments, such as *ND6* in deep-sea shrimps [38] and high-latitude loaches [39]. Therefore, positive selection of genes in this complex may have a large impact on proton pump efficiency, which affects metabolic performance. This might be related to metabolic adaptation to the stresses of mudflat habitats such as unstable oxygen, temperature, and low, often fluctuating moisture levels in terrestrial gobies. We also detected significant signals of positive selection in COIII, a component of OXPHOS complex IV that is encoded by a mitochondrial gene. OXPHOS complex IV appears to play a more vital role in energy supply compared with other complexes. Physiological studies have found that the free energy supply of complex IV is twice as high compared with that of complex I and complex III [40]. Several lines of evidence suggest that adaptive changes in the structure and activity of cytochrome c oxidase IV might enhance resistance to physiological stress in vertebrates that inhabit adverse environments [39,41]. This can, to some extent, explain why we detected positive selection in *COIII*, and suggests that the enhanced energy metabolism capacity of terrestrial gobies might help them cope with the stresses associated with the mudflat environment. In general, selection acting on OXPHOS genes might have affected the physicochemical properties of amino acids and the tertiary structure of proteins. For example, our analyses revealed obvious changes in the physicochemical properties of amino acids, including polarity and hydrophilicity, in branches leading to terrestrial gobies (Supplementary Table S9). These changes have affected the structure of ND2 and ND4 in complex I of the mitochondrial respiratory chain (Figure 5). Significant changes in the same direction have also been observed in other animals inhabiting harsh environments such as high-altitude [33] or deep seas environments [42]. Such changes might allow organisms to better cope with stressed conditions in extreme habitats [12]. 

In conclusion, our results provide new insights into the paraphyletic origin of Amblyopinae with respect to Oxudercinae, and suggest that these two subfamilies should be merged. New elements of TRS and episodes of positive selection in the mitogenome have occurred in the branches of the paraphyletic clades (Amblyopinae and Oxudercinae), indicating that they might play a role in adaptation to terrestrial habitats. The increased demand for energy and the need to cope with stresses associated with mudflat habitats might have induced physiological changes in these paraphyletic clades and triggered functional adaptations at the mitochondrial level. More in-depth analyses can take into account how these observed TRS and adaptively selected codons affect protein function and enhance oxygen utilization during the OXPHOS process. New genomic information including more mitochondrial and nuclear sequences is necessary for this clade in the future, which will likely reveal even more genes involved in the metabolic and structural processes essential for the colonization of the terrestrial realm.

## 4. Materials and Methods

### 4.1. DNA Samples and Sequence Determination

We sequenced the mitochondrial genomes of six species in four genera of the subfamily Amblyopinae: *Amblyotrypauchen arctocephalus* [17] (Alcock, 1890), *Ctenotrypauchen chinensis* [18], *Paratrypauchen microcephalus* [19], *Taenioides gracilis* [20], *T. anguillaris* [21], and *T.* sp. *Thailand* [10]. Specimens for these species were collected from various locations in coastal waters of China (Table 1). Muscle tissues were removed from each sample immediately after capture and stored in 100% ethanol. Genomic DNA was isolated from these samples using standard phenol-chloroform method [43] and 13 overlapping fragments of the mitochondrial genome were amplified by polymerase chain reaction (PCR) using sets of primers designed specifically for eel gobies (Supplementary Table S1). The PCR analysis was conducted in 50 uL volume containing 50 ng template DNA, 1× reaction buffer, 2.0 mM MgCl_2_, 0.2 mM dNTPs, 0.2 mM of each primer, and 4.0 U Taq DNA polymerase (Promega, Madison, WI, USA) using a PTC-200 (Bio-Rad, Hercules, CA, USA) PCR machine. The standard PCR conditions were as follows: 5 min initial denaturation at 94 °C, 40 cycles of 1 min at 94 °C for denaturation, 1 min at 45.7–54.1 °C for annealing, 1 min at 72 °C for extension, and a final extension at 72 °C for 5 min. All sets of PCR included a negative control reaction in which all reagents were included except for the template DNA. PCR products were sequenced using Sanger sequencing at Invitrogen Ltd., Shanghai, China. 

### 4.2. Sequence Analysis

The complete mitogenomes were assembled using the gene fragments sequenced for each species with CodonCode Aligner 5.1.5 (CodonCode Corporation, Dedham, MA, USA). The complete mitogenome was annotated using Sequin software (version 15.10), which is a mitogenome toolkit [44]. The boundaries of PCGs and ribosomal RNA genes were delimited using NCBI-BLAST. Transfer RNA genes and their clover leaf structures were identified using tRNAscan-SE 1.21 [45], with cut-off value set to 1 when necessary. The putative L-strand replication origin (OL) and control region were identified by sequence homology and proposed secondary structures. 

### 4.3. Phylogenetic Construction

Fifty-seven Gobioidei mitogenomes were downloaded from GenBank (https://www.ncbi.nlm.nih.gov/genbank/, accessed on 22 June 2022) for phylogenetic analysis (Supplementary Table S2). The nucleotide sequences of 13 PCGs from each mitogenome were concatenated and aligned using the CLUSTAL X program [46] for the phylogenetic analyses. The phylogenetic trees were constructed using the maximum likelihood (ML) and Bayesian inference (BI) methods in PhyML3.0 [47] and MrBayes 3.2.6 [48], respectively. For the ML tree, ModelTest 3.7 was used to identify the best-fitting model of sequence evolution with the Akaike information criterion. The GTR + I + G model was used for the concatenated nucleotide sequence alignment, and PhyML was used to infer the ML tree. The reliability of the tree topologies was evaluated using 1000 bootstrap replicates. For BI, the parameters estimated by ModelTest were used as priors in the analysis. Four Metropolis-coupled Markov chain Monte Carlo analyses were run for 2 × 10^6^ generations, and trees were sampled every 1000 generations. The first 25% of the runs were discarded as burn-in. In both analyses, two species in the same superorder Percomorpha, Perciformes (*Scalicus amiscus*), and Clupeiformes (*Alosa sapidissima*) were used as the outgroups.

### 4.4. Analysis of Selective Pressures

The nonsynonymous to synonymous ratio ω (dn/ds) indicates changes in selection pressure at the protein level. A dn/ds ratio of 1, <1, or >1 in PCGs typically indicates neutral mutations, negative (purifying) selection, and positive selection, respectively [32]. The CODEML program from PAML v4.8a [49] was used to analyze the ω (dn/ds) ratio using ML to detect positive selection on each mitochondrial gene. To determine whether selection pressures differed between terrestrial lineages and their non-terrestrial relatives, the “two-ratios” (M2) model was used, which assumes that the branches of interest (foreground) have different ω ratios from the background in the branch-specific models. We constructed likelihood ratio tests to compare the “two-ratios” with “one-ratio” (M0) models, which assumes identical ω values for all branches. χ2 tests were used to determine whether the M2 model was a significantly better fit than the M0 model with the threshold *p*-value < 0.05, which hinted at the selective pressure between the two branches. Since our phylogenetic analysis finally unveiled a paraphyletic origin of Amblyopinae with respect to Oxudercinae, we here set the paraphyletic clade of terrestrial gobies (Amblyopinae + Oxudercinae) as foreground, and the other non-terrestrial gobies within the same family of Gobionellidae as the background. Given that positive selection may act in very short episodes during the evolution of a protein and affect only a few sites along a few lineages. We also used a branch-site model to identify sites under positive selection along lineages of interest. We compared model A (model = 2, NSsites = 2, fix_omega = 0, omega = 5) against the null model (model = 2, NSsites = 2, fix_omega = 1, omega = 1). Positive selected sites with a posterior ratio greater than 95% were determined using Bayes Empirical Bayes analysis in CODEML [49].

To gain insight into the functional significance of the putatively selected sites, genes identified as positively selected in the branch-site test were analyzed in Expasy (http://web.expasy.org/protparam/, accessed on 24 July 2022) to identify significant physicochemical changes induced by the putative selection. We also constructed the crystal structure of positively selected genes and mapped selected sites onto this structure. The automated mode program (http://swissmodel.expasy.org/workspace/index, accessed on 24 July 2022) in the SWISS-MODEL server was used to predict and visualize the 3D structures of the proteins using the optimal templates.

## Figures and Tables

**Table 1:** Sampling locations of Amblyopinae species analyzed in the present study.

Species	Sampling Locality	Mitogenome Size	Accession No.
*Amblyotrypauchen arctocephalus* Alcock, 1890 [17]	South China Sea; Yangjiang, Guangdong	17,133 bp	MK541896
*Ctenotrypauchen chinensis* Steindachner, 1867 [18]	South China Sea; Guangzhou, Guangdong	16,552 bp	MK541901
*Paratrypauchen microcephalus* Bleeker, 1860 [19]	Yellow Sea; Dandong, Liaoning	17,086 bp	MK541897
*Taenioides gracilis* Cuvier, 1837 [20]	South China Sea; Haikou, Hainan	16,710 bp	MK541898
*Taenioides anguillaris* Linnaeus, 1758 [21]	East China Sea; Wenling, Zhejiang	16,973 bp	OL625024
*Taenioides sp. Thailand* Kurita, 2012 [10]	South China Sea; Haikou, Hainan	16,718 bp	MW682859

**Table 2:** The tandem repeat sequences in the control region of mitogenome in Amblyopinae and their relatives.

Species	Starting Point *	Sequences of Perfect Repeat (Length)	Number of Repeats	Sequences of Imperfect Repeat	Number of Repeats
*Amblyotrypauchen arctocephalus*	1157	AAACAGGAAAGACTCGAGCTAGGAATTGCATGCCCCAAACTATGTGTATATACATTATTACCAATGATTTCCCTTTGTAATAAAGCGCACATCATTCATTCAACCCTAAATACTTCAACAATTTTATAGCAGAAGCCTATATCTAA (146 bp)	4	AAACAGGAAAGCTTCGAGCTAGAAATTGCATGCCCCAAACTATGTGTATATACATTATTACAATGATTTC	1
*Ctenotrypauchen chinensis*	1156	GAAACAGGAAAGCCTCGAGCTAGGAACTACATGCCCCAAATTGCATGTATATACATTATTACAATAATACCAATACAACAAATTATTTTTTAAACCAACAACCCTAAAATCAGGAAACCTGTCAAATTTTTAAGACAGTTATGCCCCCCA (150 bp)	1	GAAACAGGAAAGCCTCGAGCTAAGAACTTCATGCCCCAAATTGCATGCATATACATTATTGCAATGATTCT	1
*Paratrypauchen microcephalus*	1157	AAACAGGAAAGCCTCGAGCTAGGAATTAACATGCCCCAAACTGTGAATACACACATTATTACAGTAATTCTGTTTGTAAACAACACAATTCAAAAATTCAACCCTTAAAAGTTTGTTATATTTATAGCACACCTCTTTCATAAA (144 bp)	4	AAACAGGAAAACCTCGAGCTAGAAATTAACATACCCCAAACGTGAATATACACACTATTATAATAATTT	1
*Taenioides* *gracilis*	1157	AAACAGGAAAGCCTCGAGCATTAAAATTAACTGCCCACAAATTACCACATATACATTATTGCAAAAATTGCTACATATACATTATTACAATAATTCTTTTACTTAAATAATATTTTATAAACATTCAACCCTCAAGCCCCTATCAACCCCTTCCCCCAAAAAG (163 bp)	1	AACAAAAAATCCTCGAGTATAAAAACCCACTGCCTACAAATTTCTACATATTCATCATTACAGACAG	1
*Taenioides* *anguillaris*	1156	AAAACAGGATAAGCCTCGAGCATAGGGACCACACCCCAAATTGCAACATATACATTATTGCAATAATTCTTTTACCTAAGCAGCAAACTATAGGCACCCCACCCTTAAAACTTCTATCAGCTTCTTTTACACTCTGACTTCACA (144 bp)	3	AAAACAGGATAAGCCTCGAGCATAGGGACCACACCCCAAATTGCAACATATACATTATTGCAAACAT	1
*Taenioides* sp. *Thailand*	1157	AAACAGGAAAGCCTCGAGTATTAAAACTTACTACTCATAAATTGCTACATATACATTATTACAAAAATTGCTACATATATATTATTACAATAATTCTTTTACTTAAATAATAAAATATAAACACTCAACCCTAAAACTTCTAACACCTCTTTACACCCCTAA (162 bp)	1	AAACAAAAAAGCCTCGAGTATAGAAACTTGCACCTGCAAACTGCTAAATATATATTATTACAGACAG	1
*Trypauchen* *vagina*	1152	CCCGGAAACAGGAAAGCCTCGAGTTAGGAATTATGTACCCCAAGTTACATACCTATACACTATTGCAATAATACCCACACAATAGACTATCTTTAAAAATTCAACCCTGAAAACAATATCAAATTTTTTATACATTTTAAA (141 bp)	2	CCCGGAAACAGGAAAGCCTCGAGTTAGGAATTATGTACCCCAAGTTACATACCTATACACTATTGCAATAATTCT	1
*Taenioides* *cirratus*	1156	AAAACAGGATAAGTCTCGAGCATAGGGACCACGCCCCAAATTGCAACATATACATTATTGCAATAATTCTTTTACCTAAGCAGCAAACTATAGGCACCCCACCCTTAAAACTTCTATCAGCTTCTTTTACACTCTGACTTCAC (143 bp)	3	AAAACAGGATAAGTCTCGAGCATAGGGACCACGCCCCAAATTGCAACATATACATTATTGCAAACAT	1
*Trypauchenopsis* sp.	1157	AAACAGGAAAGTCTCGAGCTAGGGACAAAACACCCCCAATTGCATAAATATACATTATTGCAATAATACTTTTATTGTTTTAATAATTCTATAATAGCGTGCCGTCAACTTACTATCATATTTTTTCCCCCGCCTAAAAGCCGTA (145 bp)	1	ATTTTTTATTGAAGAAAAGTAAAAATTCTTCAAGTGAACCTGCACCCCCACTTAACTCTTAAACATTATAATATAACTAA	1
*Odontamblyopus lacepedii*	1298	TGAAATCAAAGATTTCCAAGTTATATGTACACATTATTACAATAATTCACTTTTTTATAATTTAAAACAACTAATAAGCCCGTCAAAACAATCTTAAAGCAACCCCGATAAAACTTTATA (120 bp)	5	TGAAATCAAAGATTTCCAAGTTATATGTACACATTATTACAATAATTCACTT	1
*Odontamblyopus* sp.	1306	AAATCAAAGATTTCCAAGTTATATGTACACATTATTACAATAATTCATTTTTTTATAATTTAAAACAACTAACAAGCCCGTCCAAACAATCTTAAAGCGACCCCGATAAAACTTTATATG (120 bp)	3	AAATCAAAGATTTCCAAGTTATATGTACACATTATTACAATGATTCACTT	1
*Odontamblyopus rebecca*	1306	ATGTTAAAGATTTACAAGTTATATGCACACATTATTACAATAATTCACTTTTTTTATGATTTAAAACAATCAACAAGCCCGTCCAAATAGTCTTAAAGTAACCCAGATAAAACTTTATATA (121 bp)	3	ATGTTAAAGATTTACAAGTTATATGCACACATTATTACAATAATTCACTT	1
*Boleophthalmus pectinirostris*	1159	ACAGGAAAGTCTCGAGCAAGGGCCACATGCCCAAAGTTGTGTCAAATATATTACAATAATTCACTTATTAATATACAAAATAAAAGCACCCAACCCTTACTTAGCCCTAACACATCTCACCCTTTCCTAG (130 bp)	5	ACAGGAAAGTCTCGAGCAAGGGCCACATGCCCAGAGTTGTGTCAAATATATTATTATGATACTTACT	1
*Boleophthalmus* sp.	1159	ACAGGAAAGCCTCGAGTGAGGGCCAAAAACCCAAAGTTGTGTCAAAGATATTACAATAATTCACTTACTAATATACAAAATAAAAGACACCCAACCCCGACAGAGCCCTCACACATTTTATTGCTCCAACC (131 bp)	5	ACAGGAAAGCCTCGAGTGAGGGCCAAAAACCCAAAGTTGTGTCAAAGATACTATTATGATATTTTCT	1
*Boleophthalmus boddarti*	1152	CCCGGAAACAGGAAAGCCTCGAGCAAAGGGCACATACCCAAAGTTGTGTTAAACATATTACAATAATTCACTTGCTAATGTACAAAATAAGAGACACCCAACCCAGACTTAACCGTCACACACTTTTAATT (131 bp)	2	CCCGGAAACAGGAAAGCCTCGAGCAAAGGGCACATACCCAAAGTTGTGTTAAACATATTATTATGATTACTTTCT	1
*Scartelaos gigas*	1157	AAACAGGAAAGCCTCGAGTTAGGGACCATGTGCTAAAAATGTACACATACACATTATTACAATAATTCACTTATCACACAAAATAAAGATAGAAACATTTAACCCAGCATTTTCTATCATCTTTTTAACCCCCCTCCTTCCAACCGTGGACTTTTATTCACCCGGAACCTT (171 bp)	1	AACAAAACCTAGTCTAAAGTTAGAAACCACATGCTCCAAAATGCGTGTATTTACATTATTGCAATAGTTCACTT	1
*Oxuderces dentatus*	1189	ACCTCTCAAGTGTTTATGTACCAATTATTACAATAATTCATTTATTTGTACAAAAAATAATAAACCTCCGCCCTAAATAAACTAACAATTAAAAAATTACTATATAACTAATATCCCTCGATATAGATTTAACCAACACATGTAAATCGC (150 bp)	4	AGCT	1
*Parapocryptes serperaster*	1156	GAAACAGGAAAGCCTCGAGCTAGAAATCATGCCCCCAAGTTGCATGCACAAATTATTACAATAATTCACTTATTAACTCACCCCACCCATCTCACCCCAACCGAAAAATCCCTGCCAACTTTGAATCCCCCGACCCCTAAACTCCTAGAGTAATATTCACCGTTAACCCAAACCCCAA (178 bp)	4	GAAACAGGAAAGCCTCGAGCTAGAAATCATGCCCCCAAGTTGCACGGCCCCCCACCT	1
*Periophthalmus argentilineatus*	-----	-----	-----	-----	-----
*Periophthalmus modestus*	-----	-----	-----	-----	-----
*Periophthalmus minutus*	-----	-----	-----	-----	-----
*Periophthalmus magnuspinnatus*	-----	-----	-----	-----	-----
*Periophthalmodon schlosseri*	-----	-----	-----	-----	-----

* Starting point refers to where the tandem repeat sequences start in the aligned sequences of the control region in Amblyopinae mitogenome.

**Table 3:** Likelihood ratio tests of selective pressures on mtDNA genes in the paraphyletic clades of Amblyopinae and Oxudercinae.

Gene	Model	lnL	LRT	Parameter
*atp6*	M0	−12,288.396		ω0 = 0.036
	M2	−12,288.359	0.074	ω0 = 0.035; ω1 = 0.040
*atp8*	M0	−2884.378		ω0 = 0.116
	M2	−2884.367	0.021	ω0 = 0.116; ω1 = 0.128
*cox1*	M0	−21,414.251		ω0 = 0.011
	M2	−21,410.655	7.192 **	ω0 = 0.011; ω1 = 0.055
*cox2*	M0	−8871.766		ω0 = 0.017
	M2	−8871.123	1.285	ω0 = 0.017; ω1 = 0.038
*cox3*	M0	−10,794.094		ω0 = 0.024
	M2	−10,791.750	4.688 *	ω0 = 0.024; ω1 = 0.089
*cytb*	M0	−18,249.518		ω0 = 0.021
	M2	−18,248.658	1.719	ω0 = 0.021; ω1 = 0.080
*nad1*	M0	−15,830.249		ω0 = 0.021
	M2	−15,824.827	10.844 **	ω0 = 0.021; ω1 = 0.338
*nad2*	M0	−21,447.715		ω0 = 0.053
	M2	−21,437.677	20.076 **	ω0 = 0.053; ω1 = 999.000
*nad3*	M0	−6097.474		ω0 = 0.041
	M2	−6095.969	3.010	ω0 = 0.040; ω1 = 999.000
*nad4*	M0	−25,867.666		ω0 = 0.041
	M2	−25,862.873	9.585 **	ω0 = 0.041; ω1 = 0.198
*nad4l*	M0	−4790.108		ω0 = 0.032
	M2	−4789.945	0.325	ω0 = 0.031; ω1 = 999.000
*nad5*	M0	−33,909.683		ω0 = 0.044
	M2	−33,907.749	3.867 *	ω0 = 0.043; ω1 = 0.095
*nad6*	M0	−10,277.916		ω0 = 0.043
	M2	−10,275.634	4.564 *	ω0 = 0.043; ω1 = 0.912

Note: ** and * indicate diverged selective pressures between paraphyletic clades of Amblyopinae and Oxudercinae, and other Gobioidei species with a statistical significance of *p* value of <0.01 and <0.05, respectively, using LRT tests in branch model.

**Table 4:** CODEML analyses of selection on mitochondrial genes in the paraphyletic clades of Amblyopinae and Oxudercinae.

Gene	Model	lnL	LRT	Parameter	Positive Selected Site
*atp6*	Null model	−12,265.068		P_0_ = 0.940; P_1_ = 0.030; P_2a_ = 0.029; P_2b_ = 0.001; ω_0_ = 0.032; ω_1_ = 1.000; ω_2a_ = 1.000; ω_2b_ = 1.000	
	Model A	−12,264.833	0.471	P_0_ = 0.955; P_1_ = 0.030; P_2a_ = 0.015; P_2b_ = 0.000; ω_0_ = 0.032; ω_1_ = 1.000; ω_2a_ = 3.021; ω_2b_ = 3.021	
*atp8*	Null model	−2874.537		P_0_ = 0.842; P_1_ = 0.087; P_2a_ = 0.064; P_2b_ = 0.007; ω_0_ = 0.103; ω_1_ = 1.000; ω_2a_ = 1.000; ω_2b_ = 1.000	
	Model A	−2874.010	1.053	P_0_ = 0.860; P_1_ = 0.089; P_2a_ = 0.046; P_2b_ = 0.005; ω_0_ = 0.102; ω_1_ = 1.000; ω_2a_ = 4.512; ω_2b_ = 4.512	
*cox1*	Null model	−21,265.614		P_0_ = 0.979; P_1_ = 0.008; P_2a_ = 0.013; P_2b_ = 0.000; ω_0_ = 0.008; ω_1_ = 1.000; ω_2a_ = 1.000; ω_2b_ = 1.000	
	Model A	−21,265.614	0.000	P_0_ = 0.979; P_1_ = 0.008; P_2a_ = 0.013; P_2b_ = 0.000; ω_0_ = 0.008; ω_1_ = 1.000; ω_2a_ = 1.000; ω_2b_ = 1.000	
*cox2*	Null model	−8871.244		P_0_ = 0.975; P_1_ = 0.000; P_2a_ = 0.025; P_2b_ = 0.000; ω_0_ = 0.017; ω_1_ = 1.000; ω_2a_ = 1.000; ω_2b_ = 1.000	
	Model A	−8871.244	0.000	P_0_ = 0.975; P_1_ = 0.000; P_2a_ = 0.025; P_2b_ = 0.000; ω_0_ = 0.017; ω_1_ = 1.000; ω_2a_ = 1.000; ω_2b_ = 1.000	
*cox3*	Null model	−10,731.495		P_0_ = 0.946; P_1_ = 0.028; P_2a_ = 0.025; P_2b_ = 0.001; ω_0_ = 0.018; ω_1_ = 1.000; ω_2a_ = 1.000; ω_2b_ = 1.000	
	Model A	−10,728.961	5.067 *	P_0_ = 0.965; P_1_ = 0.029; P_2a_ = 0.006; P_2b_ = 0.000; ω_0_ = 0.018; ω_1_ = 1.000; ω_2a_ = 31.441; ω_2b_ = 31.441	162 (0.988)
*cytb*	Null model	−18,282.716		P_0_ = 0.899; P_1_ = 0.014; P_2a_ = 0.085; P_2b_ = 0.001; ω_0_ = 0.019; ω_1_ = 1.000; ω_2a_ = 1.000; ω_2b_ = 1.000	
	Model A	−18,282.716	0.000	P_0_ = 0.899; P_1_ = 0.014; P_2a_ = 0.085; P_2b_ = 0.001; ω_0_ = 0.019; ω_1_ = 1.000; ω_2a_ = 1.000; ω_2b_ = 1.000	
*nad1*	Null model	−15,810.905		P_0_ = 0.662; P_1_ = 0.004; P_2a_ = 0.331; P_2b_ = 0.002; ω_0_ = 0.020; ω_1_ = 1.000; ω_2a_ = 1.000; ω_2b_ = 1.000	
	Model A	−15,810.905	0.000	P_0_ = 0.662; P_1_ = 0.004; P_2a_ = 0.331; P_2b_ = 0.002; ω_0_ = 0.020; ω_1_ = 1.000; ω_2a_ = 1.000; ω_2b_ = 1.000	
*nad2*	Null model	−21,254.298		P_0_ = 0.327; P_1_ = 0.018; P_2a_ = 0.620; P_2b_ = 0.035; ω_0_ = 0.045; ω_1_ = 1.000; ω_2a_ = 1.000; ω_2b_ = 1.000	
	Model A	−21,248.610	11.376 **	P_0_ = 0.900; P_1_ = 0.050 P_2a_ = 0.047; P_2b_ = 0.003; ω_0_ = 0.045; ω_1_ = 1.000; ω_2a_ = 999.0; ω_2b_ = 999.0	87(0.966); 123 (0.987); 213(0.984); 220 (0.989); 296 (0.986); 303 (0.960); 312 (0.975)
*nad3*	Null model	−5981.539		P_0_ = 0.000; P_1_ = 0.000 P_2a_ = 0.915; P_2b_ = 0.085; ω_0_ = 0.023; ω_1_ = 1.000; ω_2a_ = 1.000; ω_2b_ = 1.000	
	Model A	−5981.466	0.146	P_0_ = 0.000; P_1_ = 0.000 P_2a_ = 0.915; P_2b_ = 0.085; ω_0_ = 0.023; ω_1_ = 1.000; ω_2a_ = 64.038; ω_2b_ = 64.038	
*nad4*	Null model	−25,655.501		P_0_ = 0.877; P_1_ = 0.057; P_2a_ = 0.062; P_2b_ = 0.004; ω_0_ = 0.030; ω_1_ = 1.000; ω_2a_ = 1.000; ω_2b_ = 1.000	
	Model A	−25,653.475	4.053 *	P_0_ = 0.928; P_1_ = 0.060; P_2a_ = 0.012; P_2b_ = 0.001; ω_0_ = 0.030; ω_1_ = 1.000; ω_2a_ = 11.628; ω_2b_ = 11.628	44 (0.991); 384 (0.988)
*nad4l*	Null model	−4771.271		P_0_ = 0.885; P_1_ = 0.028; P_2a_ = 0.084; P_2b_ = 0.003; ω_0_ = 0.028; ω_1_ = 1.000; ω_2a_ = 1.000; ω_2b_ = 1.000	
	Model A	−4771.271	0.000	P_0_ = 0.969; P_1_ = 0.031; P_2a_ = 0.000; P_2b_ = 0.000; ω_0_ = 0.028; ω_1_ = 1.000; ω_2a_ = 1.000; ω_2b_ = 1.000	
*nad5*	Null model	−33,633.451		P_0_ = 0.916; P_1_ = 0.052; P_2a_ = 0.030; P_2b_ = 0.002; ω_0_ = 0.033; ω_1_ = 1.000; ω_2a_ = 1.000; ω_2b_ = 1.000	
	Model A	−33,633.451	0.000	P_0_ = 0.916; P_1_ = 0.052; P_2a_ = 0.030; P_2b_ = 0.002; ω_0_ = 0.033; ω_1_ = 1.000; ω_2a_ = 1.000; ω_2b_ = 1.000	
*nad6*	Null model	−10,198.030		P_0_ = 0.785; P_1_ = 0.074; P_2a_ = 0.129; P_2b_ = 0.012; ω_0_ = 0.033; ω_1_ = 1.000; ω_2a_ = 1.000; ω_2b_ = 1.000	
	Model A	−10,195.755	4.550 *	P_0_ = 0.866; P_1_ = 0.082; P_2a_ = 0.047; P_2b_ = 0.004; ω_0_ = 0.033; ω_1_ = 1.000; ω_2a_ = 999.0; ω_2b_ = 999.0	78 (0.951)

Note: ** and * indicate positive selection in the paraphyletic clades of Amblyopinae and Oxudercinae with a statistical significance of P value of < 0.01 and < 0.05, respectively, using LRT tests in branch-site model.

## Data Availability

All mitogenome sequences data were deposited in Genbank with accession numer MK541896, MK541901, MK541897, MK541898, OL625024, and MW682859.

## Supplementary Materials

The following supporting information can be downloaded at: https://www.mdpi.com/article/10.3390/ijms24054362/s1.
