# Supplementary material for: Amblyopinae Mitogenomes Provide Novel Insights into the Paraphyletic Origin of Their Adaptation to Mudflat Habitats
Source: Int J Mol Sci. 2023 Feb 22;24(5):4362. doi: 10.3390/ijms24054362 (PMC10001788; doi:10.3390/ijms24054362)
Supplement: Supplementary file 1 [file ijms-24-04362-s001.zip › ijms-2159294-supplementary.pdf]

Supplementary Table S1. The primers used for mitogenome sequences amplification in six Amblyopinae species

Primers	Primer sequence	Temp (℃)	Amplicon size (bp)
P1	F 5’-CAGGCATTAGGCACAACC-3’	54.1	1220
	R 5’-AGGCAGTCTTGGAGTAGC-3’	49.1	
P2	F 5’-GAAATGAAACAACCCAGTGA-3’	52.9	1019
	R 5’-GGCTGTCTTGTGTCTGAA-3’	47.9	
P3	F 5’-CGAAGGTAGCGTAATCACT-3’	50.3	1201
	R 5’-CCAAGGCTTACTTCATAGGA-3’	52.4	
P4	F 5’-CTTACAACCAATCGCAGATG-3’	54.0	1286
	R 5’-TCATTGTCCTGTGAGTCAT-3’	47.1	
P5	F 5’-TGACTCCTAGCATGAATGG-3’	50.9	1386
	R 5’-GAGAAGAATCAGCGTGTGA-3’	50.7	
P6	F 5’-AGTGAGCATCCATCTACCT-3’	48.4	1399
	R 5’-TGAAGAGTGTAGCCTGAGA-3’	47.5	
P7	F 5’-GATTGTTCTAGCCAACTCCT-3’	51.0	1331
	R 5’-GAGGCATCTTCAAGTATTAGTG-3’	51.6	
P8	F 5’-GTTCCTGCCTTAGGTGTTA-3’	50.0	1302
	R 5’-GTCTCGTCATCACTGGTATA-3’	47.1	
P9	F 5’-CCGCACTTCTAATAACTTCTG-3’	52.7	1283
	R 5’-AGGTGAGTTCGGTGGAAT-3’	51.5	
P10	F 5’-CTGATCTATGAATGACTCCAAG-3’	51.6	1290
	R 5’-GCGTATGTGTCCAACTGA-3’	49.0	
P11	F 5’-GCACGCTCCATAGTCCTA-3’	51.1	1380
	R 5’-TGATATGATGCCAACACCTT-3’	53.4	
P12	F 5’-CTCTATGTCACATGATCTATCC-3’	48.6	1365
	R 5’-ATTGTCCGAGCATAAGGTT-3’	52.0	
P13	F 5’-CTCACTACTAAGCAACACAA-3’	45.7	1254
	R 5’-GAGAAGGACAACTCCAATG-3’	49.2	
P14	F 5’-CTGCCGAGATGTGAACTT-3’	50.5	1299
	R 5’-CTTTATTGCTTGCCTGGTTA-3’	53.8	
P15	F 5’-TGTACTATGCTTGCCCAAA-3’	52.3	1462
	R 5’-TTAAGGTTGTGCCTAATGC-3’	51.3	

Supplementary Table S2. The Gobioidei mitogenomes downloaded from the GenBank for phylogenetic analyses

Family	Subfamily	Species	Accession number
		Trypauchen vagina	NC016693
		Taenioides cirratus	KJ944420
	Amblyopinae	*Trypauchenopsis sp*	AP019362
		Odontamblyopus lacepedii	NC030374
		Odontamblyopus sp	KT633954
		Odontamblyopus rebecca	NC030481
		Boleophthalmus pectinirostris	NC016195
		Boleophthalmus sp	KP277118
		Boleophthalmus boddarti	NC023468
		Scartelaos gigas	NC028205
		Oxuderces dentatus	NC016194
	Oxudercinae	*Parapocryptes serperaster*	NC029455
		Periophthalmus argentilineatus	NC029368
		Periophthalmus modestus	AP019406
		Periophthalmus magnuspinnatus	NC028157
		Periophthalmus minutus	NC037073
		Periophthalmodon schlosseri	NC030766
		Sicydium altum	*NC036229*
Gobionellidae	Sicydiinae	*Sicyopterus squamosissimus*	MK496982
		Stiphodon alcedo	NC018054
		Acanthogobius hasta	NC006131
		Lophiogobius ocellicauda	NC020783
		Chaenogobius gulosus	NC027193
		Luciogobius elongatus	NC044716
		Gymnogobius petschiliensis	NC008743
		Eucyclogobius newberryi	NC028288
		Gillichthys mirabilis	NC012906
		Redigobius bikolanus	NC029320
	Gobionellinae	*Tridentiger barbatus*	NC018823
		Hemigobius hoevenii	NC032395
		Mugilogobius abei	NC023353
		Pseudogobius fulvicaudus	NC035428
		Rhinogobius brunneus	NC028435
		Stigmatogobius pleurostigma	NC045524
		Crystallogobius linearis	MN122854
		Gobiusculus flavescens	MT410909
		Pomatoschistus microps	MN122842
		Amblygobius phalaena	AP019316
Gobiidae		*Valenciennea longipinnis*	AP019326
		Lesueurigobius friesii	NC052760

Supplementary Table S2. The Gobioidei mitogenomes downloaded from the GenBank for phylogenetic analyses (continued)

Gobiidae	*Eviota prasina*	AP019337
	Gobiodon erythrospilus	AP019347
	Asterropteryx semipunctata	AP019328
	Callogobius okinawae	AP019318
	Amoya chusanensis	NC020347
	Exyrias puntang	NC037143
	Yongeichthys criniger	NC029234
	Favonigobius gymnauchen	NC047227
	Cryptocentrus cinctus	MT199211
	Myersina macrostoma	AP019360
	Bathygobius coalitus	NC037141
	Glossogobius circumspectus	NC018824
	Priolepis latifascima	AP019343
Butidae	*Oxyeleotris marmorata*	KJ595342
Eleotridae	*Mogurnda adspersa*	NC024058
Odontobutidae	*Odontobutis sinensis*	MZ265331
Rhyacichthyidae	*Rhyacichthys aspro*	AP004454
Outgroup 1	*Satyrichthys amiscus*	AP004441
Outgroup 2	*Alosa sapidissima*	NC014690

Supplementary Table S3. The gene organization and characteristics of mitogenome in *Taenioides anguillaris*

Gene	Position		Length (bp)	Amino acid	Codon		Anticodon	Strand
	Start	End			Start	Stop		
tRNA-Phe	1	68	68				GAA	H
12S rRNA	69	1017	949					H
tRNA-Val	1017	1088	72				TAC	H
16S rRNA	1089	2775	1687					H
tRNA-Leu	2776	2849	74				TAA	H
ND1	2850	3824	975	324	ATG	TAG		H
tRNA-Ile	3829	3898	70				GAT	H
tRNA-Gln	3898	3968	71				TTG	L
tRNA-Met	3968	4036	69				CAT	H
ND2	4037	5083	1047	348	ATG	TAA		H
tRNA-Trp	5084	5154	71				TCA	H
tRNA-Ala	5157	5225	69				TGC	L
tRNA-Asn	5227	5299	73				GTT	L
tRNA-Cys	5336	5400	65				GCA	L
tRNA-Tyr	5401	5471	71				GTA	L
COX1	5473	7026	1554	517	GTG	TAA		H
tRNA-Ser	7027	7097	71				TGA	L
tRNA-Asp	7101	7172	72				GTC	H
COX2	7177	7867	691	230	ATG	T--		H
tRNA-Lys	7868	7943	76				TTT	H
ATP8	7945	8109	165	54	ATG	TAG		H
ATP6	8106	8787	682	227	ATA	T--		H
COX3	8788	9571	784	261	ATG	T--		H
tRNA-Gly	9572	9643	72				TCC	H
ND3	9644	9994	349	116	ATG	TAG		H
tRNA-Arg	9993	10061	69				TCG	H
ND4L	10062	10358	297	98	ATG	TAA		H
ND4	10352	11732	1381	460	ATG	T--		H
tRNA-His	11733	11801	69				GTG	H
tRNA-Ser	11802	11869	68				GCT	H
tRNA-Leu	11874	11946	73				TAG	H
ND5	11947	13785	1839	612	ATG	TAA		H
ND6	13764	14303	540	179	ATG	AGG		L
tRNA-Glu	14304	14372	69				TTC	L
CYTB	14378	15518	1141	380	ATG	T--		H
tRNA-Thr	15519	15590	72				TGT	H
tRNA-Pro	15591	15660	70				TGG	L
D-loop	15661	16973	1313					

Supplementary Table S4. The gene organization and characteristics of mitogenome in *Taenioides.sp.Thailand*

**Gene**	**Position**		**Length(bp)**	**Amino acid**	**Codon**		**Anticodon**	**Strand**
	**Start**	**End**			**Start**	**Stop**		
tRNA-Phe	1	68	68				GAA	H
12S rRNA	69	1016	948					H
tRNA-Val	1017	1088	72				TAC	H
16S rRNA	1089	2780	1692					H
tRNA-Leu	2781	2854	74				TAA	H
ND1	2855	3829	975	324	ATG	TAA		H
tRNA-Ile	3834	3903	70				GAT	H
tRNA-Gln	3903	3973	71				TTG	L
tRNA-Met	3973	4041	69				CAT	H
ND2	4042	5088	1047	348	ATG	TAA		H
tRNA-Trp	5089	5159	71				TCA	H
tRNA-Ala	5162	5230	69				TGC	L
tRNA-Asn	5232	5304	73				GTT	L
tRNA-Cys	5341	5405	65				GCA	L
tRNA-Tyr	5406	5476	71				GTA	L
COX1	5478	7031	1554	517	GTG	TAA		H
tRNA-Ser	7032	7102	71				TGA	L
tRNA-Asp	7106	7177	72				GTC	H
COX2	7182	7872	691	230	ATG	T--		H
tRNA-Lys	7873	7948	76				TTT	H
ATP8	7950	8114	165	54	ATG	TAG		H
ATP6	8108	8793	686	228	ATG	TA-		H
COX3	8793	9577	785	261	ATG	TA-		H
tRNA-Gly	9577	9648	72				TCC	H
ND3	9649	9999	351	116	ATG	TAG		H
tRNA-Arg	9998	10066	69				TCG	H
ND4L	10067	10363	297	98	ATG	TAA		H
ND4	10357	11737	1381	460	ATG	T--		H
tRNA-His	11737	11806	69				GTG	H
tRNA-Ser	11807	11874	68				GCT	H
tRNA-Leu	11878	11950	73				TAG	H
ND5	11951	13789	1839	612	ATG	TAA		H
ND6	13786	14310	525	174	ATG	TAA		L
tRNA-Glu	14308	14376	69				TTC	L
CYTB	14382	15522	1141	380	ATG	T--		H
tRNA-Thr	15523	15594	72				TGT	H
tRNA-Pro	15595	15664	70				TGG	L
D-loop	15665	16710	1048					

Supplementary Table S5. The gene organization and characteristics of mitogenome in *Taenioides gracilis*

Gene	Position		Length (bp)	Amino acid	Codon		Anticodon	Strand
	Start	End			Start	Stop		
tRNA-Phe	1	68	68				GAA	H
12S rRNA	69	1016	948					H
tRNA-Val	1017	1088	72				TAC	H
16S rRNA	1089	2780	1692					H
tRNA-Leu	2781	2854	74				TAA	H
ND1	2855	3829	975	325	ATG	TAA		H
tRNA-Ile	3834	3903	70				GAT	H
tRNA-Gln	3903	3973	71				TTG	L
tRNA-Met	3973	4041	69				CAT	H
ND2	4042	5088	1047	349	ATG	TAA		H
tRNA-Trp	5090	5160	71				TCA	H
tRNA-Ala	5163	5231	69				TGC	L
tRNA-Asn	5233	5305	73				GTT	L
tRNA-Cys	5342	5406	65				GCA	L
tRNA-Tyr	5407	5477	71				GTA	L
COX1	5479	7032	1554	518	GTG	TAA		H
tRNA-Ser	7033	7103	71				TGA	L
tRNA-Asp	7107	7178	72				GTC	H
COX2	7183	7873	691	230	ATG	T--		H
tRNA-Lys	7874	7949	76				TTT	H
ATP8	7951	8115	165	55	ATG	TAG		H
ATP6	8109	8793	685	228	ATG	T--		H
COX3	8794	9577	784	261	ATG	T--		H
tRNA-Gly	9578	9649	72				TCC	H
ND3	9650	10000	351	117	ATG	TAG		H
tRNA-Arg	9999	10067	69				TCG	H
ND4L	10068	10364	297	99	ATG	TAA		H
ND4	10358	11738	1381	460	ATG	T--		H
tRNA-His	11739	11807	69				GTG	H
tRNA-Ser	11808	11875	68				GCT	H
tRNA-Leu	11879	11951	73				TAG	H
ND5	11952	13790	1839	613	ATG	TAA		H
ND6	13787	14308	522	174	ATG	TAA		L
tRNA-Glu	14309	14377	69				TTC	L
CYTB	14383	15523	1141	380	ATG	T--		H
tRNA-Thr	15524	15595	72				TGT	H
tRNA-Pro	15597	15666	70				TGG	L
D-loop	15667	16718	1052					

Supplementary Table S6. The gene organization and characteristics of mitogenome in *Amblyotrypauchen arctocephalus*

Gene	Position		Length (bp)	Amino acid	Codon		Anticodon	Strand
	Start	End			Start	Stop		
tRNA-Phe	1	68	68				GAA	H
12S rRNA	69	1015	947					H
tRNA-Val	1016	1087	72				TAC	H
16S rRNA	1088	2773	1686					H
tRNA-Leu	2774	2847	74				TAA	H
ND1	2848	3822	975	325	ATG	TAA		H
tRNA-Ile	3827	3896	70				GAT	H
tRNA-Gln	3896	3966	71				TTG	L
tRNA-Met	3966	4034	69				CAT	H
ND2	4035	5081	1047	349	ATG	TAA		H
tRNA-Trp	5082	5152	71				TCA	H
tRNA-Ala	5155	5223	69				TGC	L
tRNA-Asn	5225	5297	73				GTT	L
tRNA-Cys	5333	5396	64				GCA	L
tRNA-Tyr	5397	5467	71				GTA	L
COX1	5469	7022	1554	518	GTG	TAA		H
tRNA-Ser	7023	7093	71				TGA	L
tRNA-Asp	7097	7168	72				GTC	H
COX2	7173	7863	691	230	ATG	T--		H
tRNA-Lys	7864	7939	76				TTT	H
ATP8	7941	8105	165	55	ATG	TAG		H
ATP6	8099	8784	686	228	ATG	TA-		H
COX3	8784	9567	784	261	ATG	T		H
tRNA-Gly	9568	9640	73				TCC	H
ND3	9641	9991	351	117	ATG	TAG		H
tRNA-Arg	9990	10058	69				TCG	H
ND4L	10059	10355	297	99	ATG	TAA		H
ND4	10349	11729	1381	460	ATG	T--		H
tRNA-His	11730	11798	69				GTG	H
tRNA-Ser	11799	11866	68				GCT	H
tRNA-Leu	11870	11942	73				TAG	H
ND5	11943	1378	1839	613	ATG	TAA		H

		1						
ND6	13778	14299	522	174	ATG	TAA		L
tRNA-Glu	14300	14368	69				TTC	L
CYTB	14374	15514	1141	380	ATG	T--		H
tRNA-Thr	15515	15586	72				TGT	H
tRNA-Pro	15587	15656	70				TGG	L
D-loop	15657	17133	1477					

Supplementary Table S7. The gene organization and characteristics of mitogenome in *Ctenotrypauchen chinensis*

Gene	Position		Length (bp)	Amino acid	Codon		Anticodon	Strand
	Start	End			Start	Stop		
tRNA-Phe	1	68	68				GAA	H
12S rRNA	69	1016	948					H
tRNA-Val	1017	1088	72				TAC	H
16S rRNA	1089	2777	1689					H
tRNA-Leu	2778	2851	74				TAA	H
ND1	2852	3826	975	325	ATG	TAA		H
tRNA-Ile	3830	3899	70				GAT	H
tRNA-Gln	3899	3969	71				TTG	L
tRNA-Met	3969	4037	69				CAT	H
ND2	4038	5084	1047	349	ATG	TAG		H
tRNA-Trp	5085	5155	71				TCA	H
tRNA-Ala	5157	5225	69				TGC	L
tRNA-Asn	5227	5299	73				GTT	L
tRNA-Cys	5335	5399	65				GCA	L
tRNA-Tyr	5400	5470	71				GTA	L
COI	5472	7025	1554	518	GTG	TAA		H
tRNA-Ser	7026	7096	71				TGA	L
tRNA-Asp	7100	7171	72				GTC	H
COII	7176	7866	691	230	ATG	T--		H
tRNA-Lys	7867	7942	76				TTT	H
ATP8	7944	8108	165	55	ATG	TAG		H
ATP6	8102	8786	685	228	ATG	T--		H
COIII	8787	9571	785	261	ATG	TA-		H
tRNA-Gly	9571	9642	72				TCC	H
ND3	9640	9993	354	118	ATA	TAG		H

tRNA-Arg	9992	10060	69				TCG	H
ND4L	10061	10357	297	99	ATG	TAA		H
ND4	10351	11736	1386	462	ATG			H
tRNA-His	11732	11800	69				GTG	H
tRNA-Ser	11801	11868	68				GCT	H
tRNA-Leu	11872	11944	73				TAG	H
ND5	11945	13783	1839	613	ATG	TAG		H
ND6	13780	14301	522	174	ATG	TAA		L
tRNA-Glu	14302	14370	69				TTC	L
CYTB	14376	15516	1141	380	ATG	T--		H
tRNA-Thr	15517	15588	72				TGT	H
tRNA-Pro	15590	15659	70				TGG	L
D-loop	15660	16552	893					

Supplementary Table S8. The gene organization and characteristics of mitogenome in *Paratrypauchen microcephalus*

Gene	Position		Length (bp)	Amino acid	Codon		Anticodon	Strand
	Start	End			Start	Stop		
tRNA-Phe	1	68	68				GAA	H
12S rRNA	69	1013	945					H
tRNA-Val	1014	1085	72				TAC	H
16S rRNA	1086	2772	1687					H
tRNA-Leu	2773	2846	74				TAA	H
ND1	2847	3821	975	325	ATG	TAA		H
tRNA-Ile	3826	3895	70				GAT	H
tRNA-Gln	3895	3965	71					L
tRNA-Met	3965	4033	69				CAT	H
ND2	4034	5080	1047	349	ATG	TAA		H
tRNA-Trp	5080	5150	71				TCA	H
tRNA-Ala	5153	5221	69				TGC	L
tRNA-Asn	5223	5295	73				GTT	L
tRNA-Cys	5331	5395	65				GCA	L
tRNA-Tyr	5396	5466	71				GTA	L
COX1	5468	7021	1554	518	GTG	TAA		H
tRNA-Ser	7022	7092	71				TGA	L
tRNA-Asp	7096	7167	72				GTC	H
COX2	7172	7862	691	230	ATG	T--		H
tRNA-Lys	7863	7938	76				TTT	H
ATP8	7940	8104	165	55	ATG	TAG		H
ATP6	8101	8783	683	227	ATA	TA-		H
COX3	8783	9567	785	261	ATG	TA-		H
tRNA-Gly	9567	9638	72				TCC	H

ND3	9636	9989	354	118	ATA	TAG		H
tRNA-Arg	9988	10056	69				TCG	H
ND4L	10057	10353	297	99	ATG	TAA		H
ND4	10347	11727	1381	460	ATG	T--		H
tRNA-His	11728	11796	69				GTG	H
tRNA-Ser	11797	11864	68				GCT	H
tRNA-Leu	11869	11941	73				TAG	H
ND5	11942	13780	1839	613	ATG	TAA		H
ND6	13777	14298	522	174	ATG	TAA		L
tRNA-Glu	14299	14367	69				TTC	L
CYTB	14373	15513	1141	380	ATG	T--		H
tRNA-Thr	15514	15585	72				TGT	H
tRNA-Pro	15586	15655	70				TGG	L
D-loop	15656	17086	1431					

Supplementary Table S9. The physicochemical properties changes in amino acids in paraphyletic clades of Amblyopinae and Oxudercinae

Gene	Codon	Amino Acid change	Polarity	Charges	Hydrophobicity
cox3	162	Gly=>Ser	polar=>polar	neutral=>neutral	hydrophilic=>hydrophilic
	87	Gln=>Thr	polar=>polar	neutral=>neutral	hydrophilic=>hydrophilic
	123	Asp=>Thr	polar=>polar	**minus=>neutral**	hydrophilic=>hydrophilic
	213	Ser=>Ala	**polar=>nonpolar**	neutral=>neutral	**hydrophilic=>hydrophobic**
nad2	220	Phe=>Asn	**nonpolar=>polar**	neutral=>neutral	**hydrophobic=>hydrophilic**
	296	Ser=>Cys	polar=>polar	neutral=>neutral	hydrophilic=>hydrophilic
	303	Ile=>Met	**nonpolar=>nonpolar**	neutral=>neutral	**hydrophobic=>hydrophobic**
	312	Thr=>Ser	polar=>polar	neutral=>neutral	hydrophilic=>hydrophilic
nad4	44	Asn=>Ser	polar=>polar	neutral=>neutral	hydrophilic=>hydrophilic
	384	Thr=>Val	**polar=>nonpolar**	neutral=>neutral	**hydrophilic=>hydrophobic**
nad6	78	Tyr=>Phe	**polar=>nonpolar**	neutral=>neutral	**hydrophilic=>hydrophobic**
